# Assessment of neonatal referral infrastructure and clinical characteristics of referred neonates in three first referral hospitals in Nairobi County, Kenya

**DOI:** 10.12688/wellcomeopenres.18871.3

**Published:** 2023-11-24

**Authors:** John Wainaina, Grace Irimu, Mike English, Emily Mbaire, Mary Waiyego, Christine Manyasi, David Kimutai, Caren Emadau, Celia Muturi, Jalemba Aluvaala

**Affiliations:** 1 Health Services Unit, KEMRI - Wellcome Trust Research Programme, Nairobi, Kenya; 2 Department of Paediatrics and Child Health, University of Nairobi, Nairobi, Kenya; 3 Nuffield Department of Clinical Medicine, University of Oxford, Oxfordshire, UK; 4 Pumwani Maternity Hospital, Nairobi County, Kenya; 5 Kenyatta National Hospital, Nairobi County, Kenya; 6 Mbagathi County Hospital, Nairobi County, Kenya; 7 Mama Lucy Kibaki Hospital, Nairobi County, Kenya

**Keywords:** Newborn, referral, inter-facility, transport, inpatient care, Kenya

## Abstract

**Background:**

One in five newborns in Nairobi County, Kenya, may require inpatient neonatal care. We sought to examine referrals to and from three busy first-level referral public hospitals in Nairobi and what infrastructure and systems are available to support neonatal transport from these first-referral level hospitals to the main tertiary care center.

**Methods:**

Patient-level data of newborns over 12 months were retrospectively extracted from routinely collected patient data and examined to characterize those referred into and out of three newborn units in the study hospitals. Structural assessments using a checklist completed during hospital visits were used to describe hospitals’ readiness to support newborn referral and transport.

**Results:**

Five percent (398/7720) of the cohort studied were either referrals into study hospitals (68%, 272/398) or referrals out (32%, 126/398). Among 397 (99%) and 268 (67%) with sex and gestation documented respectively, 63% (251) were male and 44% (118) were preterm infants (<37 weeks). Among those referred in, 26% (69/272) died and 2.6% (7/272) were further referred to a tertiary-care newborn unit. Prematurity (39%) and birth asphyxia (29%) were the main in-referral reasons from 38 different health facilities, with specialist reviews (34%) predominant for out-referrals to a tertiary center. Diverse transport methods were used for referrals to study hospitals including private and public ambulances, vehicles, and guardian’s arms while onward referrals to the tertiary center were done by hospital ambulances. Drugs and medical supplies required for stabilization were well available at the study sites, however, only oxygen nasal cannula, nasal prongs, and face masks were available in ambulance of hospital 3.

**Conclusion:**

There is a need to develop, equip and maintain a high-quality referral and newborn transport system that can support the continuum of newborn care across referral care pathways into and from first-referral level hospitals.

## Introduction

To avert preventable neonatal deaths, health systems must develop the capacity for referral and safe transport of small and sick newborns (SSNB) when required ^1, 2^. About 3% of newborns admitted in primary and secondary newborn units require onward referral to tertiary newborn centers for reasons such as complications of preterm birth ^3, 4^. This is in line with the World Health Organization’s (WHO) guidelines that every SSNB with a condition that cannot be managed adequately with available resources gets an appropriate and timely referral within integrated newborn service pathways ^5^. Such services are therefore essential to a setting like Nairobi County, Kenya, where only 50% of sick newborns are estimated to have access to facilities with a capacity to deliver intermediate-level newborn care ^6^.

Neonatal transport is a key component of a referral system, though in Low and Middle-Income Countries (LMICs) it is underdeveloped and inadequate for maintaining thermal stability ^1, 7^. Further, evidence shows the current inability to provide urgent, condition-specific care such as for respiratory distress, coupled with poor adherence to neonatal transport guidelines and standards in LMICs ^1, 7^. This results in high morbidity and mortality among sick newborns transported between facilities ^1, 8^. While local guidelines and policies incorporating international standards are key to providing a roadmap for implementation, to the best of our knowledge, The Kenya Health Sector Referral Strategy does not provide any specific guidance on neonatal referral and transport ^9^.

To understand the existing capacity of the neonatal referral system of Nairobi County, a high-mortality urban setting, we sought to, (i) describe the clinical characteristics of newborns referred into and out of three busy first referral-level hospitals, (ii) identify equipment and medical supplies available for pre-transport stabilization in these facilities, (iii) describe referral communication infrastructure and finally, (iv) describe equipment, medical supplies, and human resources available for neonatal transport in the three study hospitals within Nairobi County.

## Methods

### Study sites

**Figure 1.**
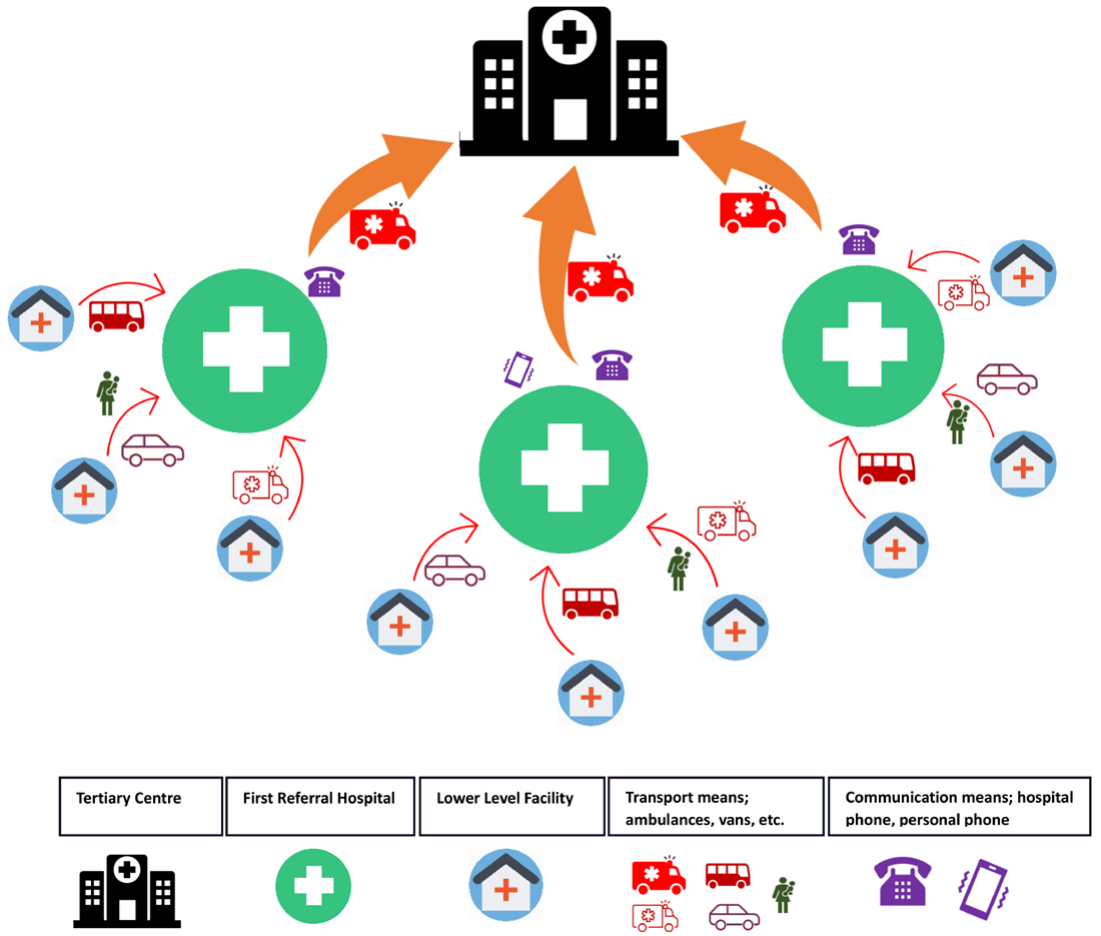
Schematic showing the relationship between the study sites, tertiary center, and lower level facilities, and the referral pathways.

This study was conducted in three public first-referral hospitals (Hospital 1, Hospital 2, and Hospital 3) in Nairobi County. These three hospitals were chosen purposively since they are among four public hospitals that offer 71% of existing 24/7 inpatient neonatal care in Nairobi County, Kenya’s capital city ^6^. The fourth hospital, a public tertiary hospital that is the destination for most public-sector referrals was excluded because it does not refer patients onward ^6^. Figure 1 shows the schematic representation illustrating the relationships between the three study sites as first-level referral hospitals, the tertiary center, and lower-level facilities as sources of referral to the first-level referral centers. This schematic aims to visually elucidate the intricate network of interactions that shape the flow of newborn referrals within this context’s healthcare system. ( Figure 1)

Hospital 1 is a maternity hospital while Hospitals 2 and 3 are general hospitals aspiring to provide 24/7 intermediate-level neonatal care (including Continuous Positive Airway Pressure (CPAP) but excluding mechanical ventilation) ^10^. They are also members of a Clinical Information Network (CIN) that aims at improving the quality of patients’ data documentation and their utilization ^11– 14^. CIN data collection procedures are described elsewhere ^11^. In brief, the trained data clerk captures routine inpatient newborn care data at the point of discharge for all patients admitted to the Newborn Units (NBUs) ^11, 14^. Data are collected mainly from structured newborn admission records (NARs) and discharge summary forms. These data are entered into a pre-designed Research Electronic Data Capture (REDCap) data collection tool, pre-programmed with field validation rules (out of range and data type) to ensure quality during entries ^11, 14, 15^. R programming scripts are also run against the database to check for errors and support data quality assurance ^11, 15^.

### Study design

This was a cross-sectional descriptive study with two major components; a) a description of clinical characteristics of referred patients and b) a structural assessment of the availability of referral infrastructure.

### Sampling and sample size

To describe the clinical characteristics and outcomes of referred newborns, we obtained 12 months (February 2018 to January 2019) of data on all newborns admitted to the study sites (N= 7720) from the CIN database ^14, 15^. Participants included in the study had to be either referred into or out of the three study sites during the study period. Identification of referrals also involved the use of NBU admission registers. There existed no formal system of archiving these registers after filling up and thus we targeted the most recent period when most information was likely to be available. 

### Study procedures

**a) Clinical characteristics and outcomes of referred neonates**

Datasets for newborns admitted in the study period across three hospitals were extracted from KEMRI Wellcome Trust Programme’s CIN REDCap servers for analysis. These data included bio-information, maternal and newborn history, examination, diagnoses, and discharge data. All three hospitals’ datasets were merged for analysis. The analysis population included only those either referred in or out of the study hospitals’ NBUs. We analyzed this population to describe their characteristics disaggregated by referral status ( *in or out*) and examined morbidity patterns and mortality rates.

**b) Conducting structural assessment**

A checklist was adapted from multiple sources including i) the European Standards of Care for Newborn Health project report, ii) the Measure Evaluation Referral Systems Assessment and Monitoring Toolkit, iii) the Quality of Care Assessment tool developed for the Health Services Implementation Research and Clinical Excellence Collaboration with the Ministry of Health in Kenya (SIRCLE), iv) International Health Facility Assessment Network (IHFAN) for Rapid Service Provision Assessment and v) other literature from unindexed databases ^16– 19^.

Although the checklist had not been previously validated in this context, we pragmatically focused on the availability of resources needed for the provision of basic referral care for small and sick newborns. This checklist was deployed into an Android tablet-based REDCap tool. A walk-through of these facilities was conducted, during which the checklist questions were posed to the nursing officers in charge. This approach involved both structured observations to evaluate the infrastructure in the newborn units and ambulances and structured interviews with the nursing officers in charge to confirm the physical presence or absence of checklist items over two weeks in March 2019. A piece of equipment was recorded as available if physically present by observation and functional.

### Data analysis

Patient-level care data were reported as frequencies and proportions of admissions and deaths disaggregated by either referrals in or out of the study sites.

Structural data were analyzed descriptively in two categories: a) pre-transport drugs, equipment, and medical supplies, and b) ambulance drugs, equipment, and medical supplies. Results were presented using a table of ticks (√) and cross (×) indicating ‘Available’ and ‘Not Available’ respectively. All analyses were done using R software.

### Ethical considerations

Ethical approval for this work was provided by KEMRI’s Scientific and Ethical Review Unit (KEMRI/RES/7/3/1 SSC PROTOCOL No. 2465).

## Results

There were 7720 admissions in the three hospitals during the study period (February 2018 to January 2019). Each hospital had at least one ambulance and at least one pediatrician. Only one hospital (Hospital 1, maternity hospital) had staff trained in neonatal transport. None of the three hospitals had staff specific for neonatal transport. Table 1 shows details about these hospitals.

**Table 1.** Study hospital and NBU details.

	Hospital 1	Hospital 2	Hospital 3
**Hospital level**			
Type	Maternity Hospital	General Hospital	General Hospital
Catchment population	~0.5 million	~2 million	~3.1 million
No. of ambulances	2	1	1
**NBU level**			
Annual NBU Admissions	4758	2057	905
No. of Referrals In	190	33	49
No. of Referrals Out to Tertiary Hospital	45	45	36
Cots	50	17	7
Incubators	11	7	10
Pediatricians	5 *	1	2
Medical Officers	5	1	1
Nurses	23	22	11
Clinical officers ^%^	4	0	2
No. trained on neonatal transport ^&^	1	0	0
No. of neonatal transport staff	0	0	0

* Includes one Neonatologist ^%^ Clinical Officer – A clinician with a minimum qualification of a Diploma in Clinical Medicine and Surgery from any accredited institution in Kenya ^&^ One specialist (neonatologist) trained in neonatal transport but involved only in NBU’s clinical services

### Neonatal referrals and their characteristics

Analyses were done on cases with information available and thus, denominators varied across indicators. Over the 7720 one-year neonatal admissions, 398 (5%) were referred. A majority (68%, 272/398) were referred to study hospitals from 38 different health facilities, and 32% (126/398) were referred outward from study hospitals to a tertiary hospital. No newborns were reported to have been referred to lower-level facilities from these three NBUs. Almost all records (99%, 397/398) had data on patient sex available, and among both referrals in and referrals outward males were the majority at 63% (251/397).

Among those referred to the study hospitals, 99% (268/272) and 75% (203/272 had birth weight and gestation age documented respectively. Less than half (44%, 118/268) were low birth weight (< 2500g) and 40% (83/203) were below 37 weeks gestation (preterm). Temperature and pulse oximetry at admission data were available in 27% (74/272) and 29% (78/272) neonatal admissions respectively. Almost half, 45% (33/74) had hypothermia on arrival (< 36.5°C) and 46% (36/78) had oxygen saturation levels below 90% at the point of admission. Most of the newborns referred to the study hospitals (74%, 201/272) were admitted on the day of birth. Among the 272 referred in, 69 (26%) died while 7 (2.6%) got referred onward to a tertiary center. Table 2 shows the neonatal characteristics of referred neonates. Among the 126 cases referred outward from the study hospitals to a tertiary facility, the ultimate outcomes were undetermined due to the absence of a comprehensive tracking and reporting system.

**Table 2.** Characteristics of referred newborns.

Characteristic	Documented, N	Referred In, n(%)
**Birth Weight Category**	268 (98.5%)	
*< 1000*		9 (3.4%)
*1000–1499*		39 (15%)
*1500–1999*		44 (16%)
*2000–2499*		26 (9.7%)
*2500–4000*		141 (53%)
*> 4000*		9 (3.4%)
*Missing*		*4 (1.5%)*
**Temperature at Admission**	74 (27.2%)	
*< 32*		0 (0%)
*32–35.9*		17 (23%)
*36–36.4*		16 (22%)
*36.5–37.5*		28 (38%)
*> 37.5*		13 (18%)
*Missing*		*198 (72.8%)*
**Oxygen Saturation at** **Admission**	78 (26.7%)	
*< 90*		36 (46%)
*≥ 90*		42 (54%)
*Missing*		*194 (71.3%)*
***Outcome***	268 (98.5%)	
*Alive*		192 (71%)
*Dead*		69 (25%)
*Referred Out*		7 (2.6%)
*Absconded*		0 (0%)
*Missing*		*4 (1.5%)*

**Figure 2.**
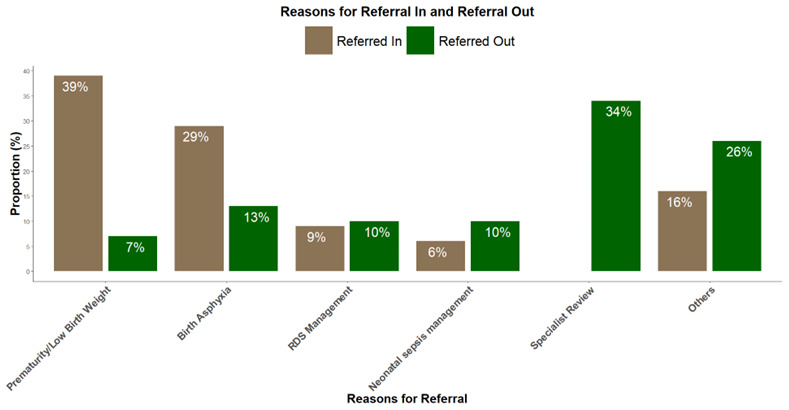
Shows reasons for referral in and out of study hospitals. ***Other reasons for referrals in included*** chorioamnionitis, refusal to breastfeed, macrosomia, difficulty breathing, cyanosis, jaundice, maternal condition, etc. ***Other reasons for referrals out included*** Electrocardiography, congenital malformations, advanced airway support, etc.

Figure 2 shows reasons for referral in and out of study hospitals.

### Neonatal pre-transport stabilization equipment and medical supplies

Hospitals were moderately well-resourced to stabilize and manage babies referred to the facility. 32 of 49 items (equipment and drugs) required for stabilization care were available in all three study hospitals. These included radiant warmers, heating sources, suction machines, gentamicin, crystalline benzylpenicillin, and vitamin K among others. One of the hospitals was missing some items such as an emergency area wall clock, wall thermometer, stabilization guidelines, and resuscitation checklists. Table 3 shows the per-hospital availability of indicators required for stabilization and pre-referral newborn care. Consumables for different devices such as incubators, and radiant warmers among others were not assessed.

**Table 3.** Indicators required for stabilization and pre-referral newborn care.

Indicator	Hospital 1	Hospital 2	Hospital 3	Indicator	Hospital 1	Hospital 2	Hospital 3
Stabilization Guidelines	√	√	×	Radiant warmer	√	√	√
Resuscitation Checklist	√	√	×	Heat Source	√	√	√
Dosage Guidelines	√	√	√	Suction Machine	√	√	√
Pulse Oximetry Guide	√	×	×	Suction Catheters	√	√	√
Jaundice Management Guide	√	√	×	Bag size 500ml	√	√	√
Gestation Age Estimation Chart	√	√	√	Mask 0.1.2	√	√	√
Incubator Temperature Setting	×	√	×	Reservoir Bag	√	√	√
Vitamin K	√	√	√	Pulse Oximeter	√	√	√
1% TEO ^†^	√	√	√	Sterile Cord Clamp	√	√	√
Nevirapine Solution	√	√	√	Emergency Area Wall Clock	√	√	×
Benzyl Penicillin	√	√	√	Oxygen Source	√	√	√
Gentamicin	√	√	√	Intravenous Fluid(IVF) Giving Set	√	×	×
Ampicillin Injection	√	×	×	Blood Transfusion Set	√	√	√
Metronidazole Injection	√	√	√	3-Way Catheter	×	√	√
Oral Amoxicillin	√	×	√	Cannula Scalp Vein Sets	√	√	√
Oral ampicillin	×	×	×	Nasal Gastric (NG) Tubes	√	×	√
Phenobarbitone Injection	√	√	√	Needles	√	√	√
Phenytoin Injection	√	√	√	Syringes	√	√	√
				Normal Saline	√	√	√
Ceftriaxone	√	×	√	IV Solutions ^ð^	√	√	√
				Antiseptic Solution	√	√	√
				Wall Thermometer	√	√	×
Cefotaxime	√	×	×	Normal Thermometer	√	√	√
				Low Reading Thermometer	×	√	√
				Examination Light	√	√	√
Amikacin	√	√	×	Digital Weighing Scale	√	√	√
				Dextrose 10% (D10W)	√	√	√

^†^ 1% Tetracycline Eye Ointment/TEO ^ð^ Intravenous solutions: potassium chloride(KCL), ringers lactate

### Referral documentation and communication infrastructure

The three study hospitals had client outward referral forms, whose structure and contents differed. They all had phone contacts of the hospital’s ambulance and those of the newborn unit at the tertiary hospital to support outward referrals. Contacts of lower-level facilities were not available. No hospital had a patient transit care and monitoring form to document care given to the patient while *en route* as an outward referral. One newborn unit did not have a referral services phone, nor a referral register for documenting both referrals in and outward events details such as the name of the referring facility and reasons for referral. Nurses would use their mobile phones to make referral communication with the tertiary hospital’s NBU. Referrals in and outward services audit and evaluation documents were entirely unavailable across the three hospitals, including client feedback forms, referral indicators forms, and facility referral summaries among others. Further, there did not exist any e-referral facilities. Table 4 (A) shows the state of availability of referral information and communication resources.

**Table 4.** (A) Indicators for Referral Information and Communication and (B) Transport elements (In the Hospital Ambulance).

A) Indicators for referral information & communication				B) Transport elements (In the Hospital Ambulance)			
Indicator	Hospital 1	Hospital 2	Hospital 3	Indicator	Hospital 1	Hospital 2	Hospital 3
Directory of Facilities	√	√	√	Specialist	×	×	×
Directory of Services	√	×	×	Medical Doctor	×	×	×
Referral in Register	√	√	×	Registered Nurse	√	√	√
Referral Out Register	√	×	√	Clinical Officer	×	×	×
Patient Referral form	√	√	√	Respiratory Therapist	×	×	×
Specimen Referral Form	√	√	√	Paramedic	×	×	×
Consultation Form	√	×	×	Driver	√	√	√
Patient Feedback Form	×	×	×	Transport Resuscitation Guidelines	×	×	×
Referral Evaluation Report	×	×	×	Transport Stabilization Guidelines	×	×	×
Transport Training Report	×	×	×	Thermoregulation Guidelines	×	×	×
Referral Indicator Forms	×	×	×	Reintubation on Transit	×	×	×
Transit Monitoring Form	×	×	×	Transport Phenobarb Injection	×	×	×
Facility Referral Summary	×	×	×	Transport Mobile Incubator	×	×	×
Referral Report	×	×	×	Oxygen Nasal Cannula	×	×	√
Back Transfer Register	×	×	×	Nasal Prongs	×	×	√
Referral Phone	√	√	×	Face Mask	×	×	√
				Transport Monitor	×	×	×
Ambulance Team Contacts	√	√	√	Transport Pulse Oximeter	×	×	×
				BP Measuring Device	×	×	×
Contacts Other Hospitals	√	√	√	Intubation Equipment	×	×	×
				Airway Support Equipment	×	×	×
E-referral Infrastructure	No	No	No	Suction Machine	×	×	×
				Stethoscopes	×	×	×
Referral Focal Person	√	√	√	Thermometer	×	×	×
				Backup Battery	×	×	×

### Neonatal transport resources, drugs, equipment, and medical supplies in the ambulance(s)

Transport infrastructure was assessed in two components a) internal and b) external. The internal component had two dimensions: into the newborn unit (from the hospital’s labor ward and other lower-level facilities) and out of the newborn unit. Newborns arrived at first-level referral hospitals using diverse transportation methods, including various vehicles, on mother’s or guardian’s arms, as well as county and privately procured ambulances. Neonates being brought into the newborn unit and out to a higher-level hospital were carried in the mother’s or guardian’s arms and further transported in the hospital’s ambulance to a tertiary care center. No use of a mobile baby incubator was reported. A mobile oxygen source from the NBU could be carried alongside the baby to the ambulance for outward referrals. Referral back to lower-level facilities was never reported in any of the three facilities.

All three hospitals had one NBU, and none had a dedicated neonatal ambulance but rather at least one ambulance for general hospital use. Only oxygen nasal cannula, nasal prongs, and face masks were available in the ambulance of hospital 3. Transport resuscitation guidelines, a thermoregulation guide, phenobarbitone, oxygen cylinders, pulse oximeters, and patient thermometers among others were not available in ambulances. Other than one neonatologist from one of the three hospitals, no other staff had specific training in neonatal transport. Table 4 (B) shows the state of availability of neonatal transport resources as assessed in ambulances.

During outward referral from the three hospitals, one nurse, a driver, and the mother/guardian could accompany the baby, typically to the tertiary hospital’s newborn unit. The nurse from the referring hospital could carry the baby to the newborn unit of the receiving tertiary hospital, open a new patient’s file, document nursing notes of the care received at the referring hospital, and also admit the mother in the post-natal ward of that hospital. This process was reported to take not less than six hours during which the referring hospital’s nurse was therefore away from the main workstation.

## Discussion

We sought to describe the characteristics of newborns referred into and out of three county hospitals in Nairobi County, Kenya, and the infrastructure in place to support their referral and transport to higher levels of care. Birth asphyxia and other intrapartum-related complications are the most common (29%) causes of newborn admissions. Bringing down these phenomena means a wholesome improvement in the quality of care in the antenatal period, labor management, quality child-birth services, and immediate postnatal period ^14^. Prematurity (27%) and low birth weight (16%) were other common conditions leading to admissions at the three hospitals’ NBUs. These are often complicated with RDS, morbidity that these hospitals are poorly equipped to handle, for example, none or just one continuous positive airway pressure (CPAP) machine ^14^.

About 3%–5% of admissions in the newborn units of the study hospitals were referrals. Clinical diagnoses of this referred cohort, occurring in proportions similar to those not referred, are similar to those reported in a study done in Uganda, although, mortality was lower (26% vs. 33%) (Hedstrom, A. *et al.* 2014) ^20^. Similarly, the need for specialized treatment (47.1%) was also recorded in a Tanzanian study as the top reason for referral to a tertiary center ^21^. This demonstrates that improvements should focus on making a functional health system that caters to the needs of these critically ill newborns needing transport to higher levels and those inborn ^22, 23^. As might be expected, a newborn baby would highly benefit and have the best outcomes if they are born at a facility with relevant infrastructure, equipment, and human resources expertise capable of taking care of the needs presented ^24, 25^. This is *in-utero* transfer and suggests a need to enable health facilities to transport at-risk mothers early enough to higher levels facilities ^24, 26^.

Among those referred to three study hospitals, almost half (45%) had hypothermia. This has consistently been documented as highly prevalent among transported newborns, although, with varying proportions. Vieira *et al.* 2011, Mank A. *et al.* 2016, and Alebachew B. *et al*. 2019 report hypothermia prevalence of 16%, 30%, and 66.3% respectively ^27– 29^. Notably in this study, no equipment or monitoring tools/forms for tracking temperature or other vital signs such as pulse oximetry during transport from these urban, intermediate-level NBUs were available. Newborns exposed to cold temperatures are at an increased risk for death and more among those born prematurely who are often suffering respiratory distress, a condition that makes their ability to respond to hypothermia even more difficult ^30, 31^. A high prevalence of hypothermia among transported newborns could indicate low adoption of Kangaroo Mother Care (KMC) during neonatal transport, at least for newborns who meet set criteria ^32^. With limited resources, especially in low-income countries, the adoption of such affordable, yet effective methods could help reduce hypothermia during transport and consequently reduce neonatal mortality among referred newborns ^32, 33^. This method also facilitates ‘zero separation’ benefits and family-centered care aspects in the newborn period ^33^. 

There are substantial gaps in neonatal referral and transport readiness indicating the need to establish functional systems to ensure availability and access to widespread high-quality newborn care across levels of health care ^14^. These neonatal transport system improvements will cater to newborns, especially those born at primary and first-level referral facilities helping them access lifesaving care at tertiary centers. Further, these improvements must also include capacity strengthening to deliver high-quality care to meet the demand at every level of the perinatal health system and subsequent improved neonatal survival ^8, 34^.

Communication and information systems are an integral part of Kenya's Health Sector Referral Strategy for an effective referral system aimed at making available data for decision-making, planning, investment, and accountability ^9^. This study reports some level of compliance with these requirements other than for summary reports, referral feedback forms, and channels. This gap was similarly observed in both Ethiopia and Tanzania reported huge gaps in communication and poor documentation systems for patient indicators ^35^ (Mpokigwa K. *et al.* 2022) ^21^. The systems in place in the study sites fall well below those recommended by WHO’s Standards for improving the quality of care for small and sick newborns ^5^. These require the establishment of referral coordinating centers, information exchange between referring facilities, timeliness, and adherence to clinical care guidelines and protocols customized to the standard level of care at any given point in the integrated referral care pathways ^20^.

Having a majority of medical supplies available for the stabilization of newborns referred from other facilities and early stabilization before transport for those being referred outward is key to mitigating adverse events and improving outcomes ^36^. Referral guidelines explicitly indicate that hospitals should prepare a customized list of equipment and medical products needed for emergency referral situations and set them aside in readiness for emergencies ^9^. Though a majority of these items were available within newborn units and the hospitals, none of the sites had an explicit checklist list or particular items set aside for those being referred which could be used to support the referral journey. This may cause delays and inefficiencies that would result in poor care, delayed service and ultimate loss of newborn lives ^35, 36^.

Neonatal transport readiness across study sites was poor. While neonatal transport is a critical linkage to care for babies born at peripheral facilities to centers with resources and capacity to handle their medical and surgical needs, the results of this study describe a hazardous system for newborns ^23, 37^. This might explain why 75% of babies transferred in LMICs reach tertiary centers with serious complications and in a moribund state ^38, 39^. Although not specific to newborn transport, the Kenya Health Sector Referral Strategy dictates that a health facility should maintain a necessary referral transport infrastructure that contains and adheres to minimum ambulance requirements for effective client transfer ^9^. This includes a specific team with requisite training and expertise with clearly defined roles and skills to manage and support patient transport ^40^. Our observations indicate there were no specific newborn transfer services and almost no generally qualified, experienced, or specialized transport teams working with specialist-equipped transport vehicles ^5^. Other studies have reported the absence of ambulances, and where available, that they had incomplete medical supplies including oxygen supply, warmers, and patient monitors among other essential items for newborn care during transit ^41^.

Reasons for referral were often not documented in the available registers. This means that in some instances we were unable to capture this as distinctly from the admission diagnosis. In addition, in two of the hospitals, neonates are also admitted to the general pediatrics wards. This population was not included in the analysis as our focus was on the neonatal units in these first referral-level hospitals. This study is also subject to a notable constraint due to its retrospective observational nature, which entails dependence on medical records and ward registers. This framework inherently constrains the ability to oversee data collection and may lead to instances of data gaps or incompleteness. Additionally, the reliance on these sources introduces the potential for inaccuracies or disparities in documentation. Moreover, we didn't assess the adequacy of resources against international thresholds and in relation to the number of sick newborns admitted or referred, thus we cannot comment on resource sufficiency. Taken together, these factors underscore the necessity for a meticulous interpretation of the study's findings and implications.

## Conclusion

The findings underscore the urgent need for a robust neonatal transport system to bridge newborn care accessibility and availability gaps across healthcare levels. While our study supports concerns about neonatal transport, we acknowledge its inherent limitations. Birth asphyxia and prematurity highlight the need for comprehensive strategies spanning antenatal to postnatal care. The high hypothermia rate among referred newborns emphasizes Kangaroo Mother Care integration during transport for reduced mortality. Effective communication systems are vital for referrals, while neonatal transport readiness remains suboptimal. Recognizing limitations like missing referral reasons and data disparities reinforces cautious interpretation. The Ministry of Health and other stakeholders should develop specific guidelines for newborn referral and transport, facilitating system investment cases. Addressing these limitations and robust evaluation will guide future efforts to enhance neonatal care and transport for better outcomes.

## Data Availability

Harvard Dataverse. Referral data for 2018-2019 in the neonatal arm of the Clinical Information Network (CIN-Neonatal) [DOI: 10.7910/DVN/KBMN8N] ^42^. This project contains the following underlying data: CIN-Neonatal-Referral-Dataset2018-19.tab (Patient level data describing newborn characteristics necessitating their admissions.) Data_codebook.docx (This contains the dictionary of the two datasets involved to support understanding of data types, variable names and field labels and options per variable). Readme _CIN_Referral_JWainaina.txt. (This contains details about the project, datasets, terms of data use/data access, contents, method, and processing of the data involved). Referral Readiness Facility Assessment Dataset 2019.xlsx. (Checklist developed from literature search of important items and indicators necessary for a referral system; the equipment, drugs, medical supplies, components in transport ambulance, information systems, staffing and handover). Data is available under the terms of the [ Creative Commons Attribution 4.0 International]. *The data utilized in this work was made available to the research team by the participating hospitals and the Ministry of Health, and thus for some of the data, we are not the primary data owners; our use of these routine hospital data is approved as part of a specific ethical review process. Further access to the data can be sought through a request to KEMRI Wellcome Trust Research Programme's Data Governance Committee through Data_Governance_Committee@kemri-wellcome.org*.

## Software availability

R statistical programming software scripts used in analysis of underlying data can be accessed through the [DOI: 10.6084/m9.figshare.22148771] ^43^

This project contains the following underlying files

•    Transport & Referral Analysis.Rmd (R script (Rmarkdown), pulling the two datasets from the server, cleaning, manipulating, and analyzing the two datasets involved in this manuscript. The datasets are a) Referral Readiness Facility Assessment Dataset 2019.xlsx and b) CIN-Neonatal-Referral-Dataset2018-19.tab)

•    Codebook - Neonatal Transport and Referral Infrastructure Checklist_ REDCap.pdf (A checklist codebook/dictionary that lists the items checked for in the hospital Newborn Units and the ambulances as supportive elements for safe neonatal stabilization, referral, and transport.)

•    CIN-Neonatal Codebook.xlsx (Codebook/dictionary for the tool used to routinely capture data on inpatient newborn clinical care. It contains biodata, history, examination, diagnoses, supportive care, and discharge details.)

Data are available under the terms of the Creative Commons Zero "No rights reserved" data waiver (CC0 1.0 Public domain dedication).
