# Supplementary material for: Assessment of neonatal referral infrastructure and clinical characteristics of referred neonates in three first referral hospitals in Nairobi County, Kenya
Source: Wellcome Open Res. 2023 Nov 24;8:126. Originally published 2023 Mar 21. [Version 3] doi: 10.12688/wellcomeopenres.18871.3 (PMC11538596; doi:10.12688/wellcomeopenres.18871.3)
Supplement: Peer review history

# Reviewer report: Reviewer response for version 3

**DOI:** 10.21956/wellcomeopenres.22432.r109191

**Published:** 2024-11-18

**Authors:** Hippolite O Amadi

**Affiliations:** 1 Imperial College London, London, England, UK

Page 2, ABSTRACT: Last sentence of 'Result':

 You never defined 'hospital 3'. You may choose to rewrite this as ".......of only one hospital". Alternatively, mention the hospital

 This study tried to understand the capabilities of the Nairobi regional neonatal care system with particular interest in the efficiency of patient transfers during referrals. The study is robust and acceptably methodological. The authors were able to identify the gaps that could be viewed as being responsible for any poor outcomes associated with neonates referred 'in' and 'out' of the healthcare facilities.

 Generally, this presents yet another version of the same old findings of poor, unsustainable systems that presents a 'structure on paper' but 'empty' in reality. The paper is beautifully presented and shows that Kenya is no different from other LMICs like Nigeria, with volumes of articles that count the 'woes' but with little that create or push the boundaries of sustainable local solutions.

 I commend the authors for clarifying and isolating the gaps that can make it easier for interested problem solvers to specifically target the gaps to sustainably bridge these.

 Page 6, paragraph 2: “ **Among the 272 referred in, 69 (26%) died while 7 (2.6%) got**

**referred onward to a tertiary center**.”

 You wrote "Among the 272 referred in, 69 (26%) died while 7 (2.6%) got referred onward to a tertiary center." This suggests that 7 cases were referred out. However, in a later sentence, you said "Among the 126 cases referred outward from the study hospitals to a tertiary facility, the ultimate outcomes were undetermined...".

 This is confusing and sounds to suggest that there could be a mix up of other patients that haven not been defined in this narrative. This requires clarifications.

 Page 11: **Reasons for referral were often not documented in the available registers. This means that in some instances we were unable to capture this as distinctly from the admission diagnosis. In addition, in two of the hospitals, neonates are also admitted to the general pediatrics wards. This population was not included in the analysis as our focus was on the neonatal units in these first referral-level hospitals**. [Adequate provision should have been made in the study design to capture patients in ‘the general pediatrics ward’ because of the well-known ad hoc systems of care in many LMIC care systems. This is a very serious limitation across most publications and investigative research of LMIC scholars. I commend the efforts of many LMIC scholars in investigating the dysfunctional organic methodologies of neonatal care repeatedly from all the regions. We have a full wealth of things that never worked and would never work in different versions from different places, and counting. There are endless investigations of the prevalent morbidities and the percentage share of deaths coming from these. The world is too rich with this information, but the LMIC researchers seem not to realise that correction factors, ideas and their implementations geared towards eventual change of the narrative is what globalhealth is waiting for now. This is another study buttressing the "gaps" that fuel the LMIC failures, without implementation of home-grown original ideas to change the narrative. In 100 years’ time, the LMIC researchers will continue to supply updated versions of the failures if they fail to think, design, develop, and implement now in order to save their neonates tomorrow. The ideas for new and functional technologies and procedures peculiar to their various locations are within their ability to radically develop, rather than folding their hands and waiting for the 'magic wand' of foreign thinkers who may not know much about their peculiar cultural dispositions, climate, and infrastructure that weigh heavily on the success of neonatal care. This paper is full of non-functional and organisational gaps that could habitually said to be there in principle but are never there in reality. This narrative in common across the LMICs and helps no African country to come out of the mess. It is like someone shooting himself in the foot and expecting to run faster out of trouble. My understanding is that this article suggests that there is a need for a total new operational construct that could be quantifiable and believable. Perhaps, a construct that could be initiated and progressively implemented over some years, without losing covered grounds, until the full implementation is achieved. The authors’ recommendations should be clear on this, so that our next read from Nairobi will be on implemented sustainable solution]

 Page 11: **Addressing these limitations and robust evaluation will guide future efforts to enhance neonatal care and transport for better outcomes**. [The conclusion has said the facts like other articles, too many, but lacking in decisive solutions for Africa and the LMICs to move forward on issues of poor neonatal care. Authors could recommend that: There may be need to emphasis a radical regional conference of local inventors who are passionate enough to be educated on these gaps, and then let them loose to go thinker out best region-specific solutions to end this round-the-circle repetition of the problems the literature has had decades unending.]

Is the work clearly and accurately presented and does it cite the current literature?

Yes

If applicable, is the statistical analysis and its interpretation appropriate?

Yes

Are all the source data underlying the results available to ensure full reproducibility?

Partly

Is the study design appropriate and is the work technically sound?

Yes

Are the conclusions drawn adequately supported by the results?

Yes

Are sufficient details of methods and analysis provided to allow replication by others?

Partly

Reviewer Expertise:

Technologies and LMIC neonatal care

I confirm that I have read this submission and believe that I have an appropriate level of expertise to confirm that it is of an acceptable scientific standard, however I have significant reservations, as outlined above.

# Reviewer report: Reviewer response for version 3

**DOI:** 10.21956/wellcomeopenres.22432.r106512

**Published:** 2024-11-05

**Authors:** Peter Saula

**Affiliations:** 1 School of Medicine, Moi University, Eldoret, Kenya

This manuscript covers a very important area of newborn care that underscores its scientific relevance. The  retrospective aspect provided patient-level data of transferred newborns, while direct interviews provided data on the facilities available for newborn transport. Data analysis though simple provided adequate answers to the research question. Data is clearly presented in the results. However, the conclusions made seem to be recommendations as they are not derived from the results of the study. Furthermore, there is no direct connection between the characteristics of  transferred newborns; and the state/availability of the newborn transport facilities. A few typographical errors were also noted.

Is the work clearly and accurately presented and does it cite the current literature?

Yes

If applicable, is the statistical analysis and its interpretation appropriate?

Yes

Are all the source data underlying the results available to ensure full reproducibility?

Yes

Is the study design appropriate and is the work technically sound?

Yes

Are the conclusions drawn adequately supported by the results?

Partly

Are sufficient details of methods and analysis provided to allow replication by others?

Yes

Reviewer Expertise:

Public health aspects of pediatric surgery including newborn transport; pediatric oncology; quality improvement in pediatric surgical care.

I confirm that I have read this submission and believe that I have an appropriate level of expertise to confirm that it is of an acceptable scientific standard, however I have significant reservations, as outlined above.

# Reviewer report: Reviewer response for version 3

**DOI:** 10.21956/wellcomeopenres.22432.r70510

**Published:** 2023-12-28

**Authors:** Helen Nabwera

**Affiliations:** 1 Department of Paediatrics and Child Health, The Aga Khan University, Nairobi, Kenya

I am happy that all my comments have now been addressed by the co-authors and would like to approve this manuscript. This is a very important manuscript that will inform the next steps on how we design and deliver neonatal care services in Kenya and the rest of the continent.

Is the work clearly and accurately presented and does it cite the current literature?

Yes

If applicable, is the statistical analysis and its interpretation appropriate?

Partly

Are all the source data underlying the results available to ensure full reproducibility?

Partly

Is the study design appropriate and is the work technically sound?

Yes

Are the conclusions drawn adequately supported by the results?

Partly

Are sufficient details of methods and analysis provided to allow replication by others?

Partly

Reviewer Expertise:

Infant and early child health, growth and nutrition. Currently focusing on the post-discharge care of preterm/low birthweight infants in Africa.

I confirm that I have read this submission and believe that I have an appropriate level of expertise to confirm that it is of an acceptable scientific standard.

# Reviewer report: Reviewer response for version 2

**DOI:** 10.21956/wellcomeopenres.22112.r66561

**Published:** 2023-10-09

**Authors:** Helen Nabwera

**Affiliations:** 1 Department of Paediatrics and Child Health, The Aga Khan University, Nairobi, Kenya

I would like to thank the authors for addressing most of my comments. The manuscript has improved significantly.

 Before this is published as the final version, I would request that the authors address the 2 issues I have listed below:  

1. Please proofread it again to address the remaining **grammatical and typing errors** e.g. “ *Participants included into the study” *(Sampling and Sample size) – should be “Participants included in the study…” *and randiant warmers* (Neonatal pre-transport stabilization equipment and medical supplies).
2. There was no comment on **whether the equipment listed met the international thresholds for adequacy when linked to the number of sick newborns admitted/referred**. I presume it was not possible to do this, so this needs to be stated as a limitation.

Well done for this excellent piece of work.

Is the work clearly and accurately presented and does it cite the current literature?

Yes

If applicable, is the statistical analysis and its interpretation appropriate?

Partly

Are all the source data underlying the results available to ensure full reproducibility?

Partly

Is the study design appropriate and is the work technically sound?

Yes

Are the conclusions drawn adequately supported by the results?

Partly

Are sufficient details of methods and analysis provided to allow replication by others?

Partly

Reviewer Expertise:

Infant and early child health, growth and nutrition. Currently focussing on the post-discharge care of preterm/low birthweight infants in Africa.

I confirm that I have read this submission and believe that I have an appropriate level of expertise to confirm that it is of an acceptable scientific standard, however I have significant reservations, as outlined above.

# Reviewer report: Reviewer response for version 1

**DOI:** 10.21956/wellcomeopenres.20925.r55688

**Published:** 2023-05-11

**Authors:** Helen Nabwera

**Affiliations:** 1 Department of Paediatrics and Child Health, The Aga Khan University, Nairobi, Kenya

Overall, this is a very important study that seeks to identify the health system gaps in the referral of sick newborns for care in the Nairobi County of Kenya. The background and rationale for this work are well-described. The Methods are clear but additional information is needed for an in-depth understanding of the procedures of data collection and analysis. The results are presented well with very clear tables and figures and the discussion addresses the key findings. However, this manuscript could be strengthened by addressing the following:

- In the Abstract, please provide more details about the methods used and results obtained for the conclusion to be valid.
- In the Methods, please provide more clarity on the data collection and analysis procedures.
- In the Results, please clearly outline the proportion of missing data in the tables and text.
- In the Discussion, please include the limitations of this study and how that influences your conclusions.
- In the Conclusion, it would be prudent to be less definitive about your findings and their immediate link to policy due to the limitations. Your study has generated very important preliminary data that has the potential to inform a more robust evaluation, whilst appreciating the challenges of using data collected in a routine resource-limited clinical setting.
- There are several grammatical errors that can be addressed by proofreading.
- Please define all abbreviations e.g. LMICs when you first use them.
- Please avoid switching between British/American English spelling- pick one and be consistent.

**Abstract**

***Background:***

*Almost one in five newly born babies in Nairobi County, Kenya,*

 Please use scientific terminology here that you can consistently use in the rest of the manuscript. E.g. “newborn infants” or “neonates”.

***Methods:***

 The word “abstracted” is incorrectly used here. Please replace with “extracted”.

*Structural assessments using a checklist completed during hospital visits were used to describe hospitals’ readiness to support newborn referral and transport.*

 Please provide some brief details about whether this a checklist that has been validated in this context or recommended by WHO etc.

 This should also contain some brief details about the 3 referral hospitals where these data were collected. What level of neonatal care do they provide? This will help the reader to understand why these 3 hospitals were chosen.

***Results:***

*A majority (397/398) and two-thirds (268/398) of patients had their sex and gestation age documented respectively.*

 Please revise this sentence for more clarity. E.g. The majority (397/398) had their sex documented and…

*…44% (118/268) were preterm infants.*

 Please provide a definition for “preterm infant” (<37 weeks) in brackets.

 Also, please provide more data on the common conditions that these infants who were referred apart from prematurity that you have highlighted. Please present brief statistics from the structural assessment instead of a narrative that summarises them.

***Conclusion:***

 This doesn’t align to the results that are presented. It would be useful for the authors to review what results they have that align to this conclusion. For instance, apart for the sex and gestation age of the infants there is limited information on their clinical characteristics that the title of the manuscript suggests are key to this manuscript.

**Introduction**

*Paragraph 1: Such services are therefore essential to a setting like Nairobi County, Kenya, where only 50% of Nairobi’s sick newborns are estimated to having access to….*

 Please drop the “Nairobi’s", as the sentence before provided the details of the context.

*Paragraph 2: This results in high mortality and morbidity among inter-facility transported sick newborns*

 Please revise to “This results in high morbidity and mortality among sick newborns transported between facilities.”

 It is written succinctly and the rationale is clear. However, please revise some the sentences as indicated and others that are long.

**Methods**

 Adequate details about study sites and the rationale for selecting them.

***Sampling and sample size:***

*All three public hospitals in Nairobi County that offered intermediate-level small and sick newborn care were included in the study*.

 This should refer to the infants, should it not? This first sentence is redundant as it provides information about the study sites that has already been stated. The subsequent paragraph provides the relevant details.

***Study procedures:***

*This checklist was deployed into a REDCap tool and…*

 Does this mean that you used tablets/mobile phones to collect these data? It is not clear as stated.

*…data were collected by walking through the facilities and newborn units and assessing ambulances. This involved interviews with the nursing officer in-charges of the newborn units over two weeks in March 2019.*

 It would be better to report it as “Structured observations of the infrastructure in the newborn units and ambulances were conducted (stating the number). In addition, interviews (describing the type of interviews and how these data were captured) were conducted with nursing officer in-charges of the newborn units to ascertain…..”

***Data analysis:***

 This is clearly outlined. It would be useful to describe how the data from the interviews was analysed. How did you merge it with the checklist or did you use both interviews and structured observations to complete the checklist?

**Results**

**Table 1:**

 In addition to listing the number of incubators in each hospital, it would be useful to state how many are functional.

***Neonatal referrals and characteristics***

 It would be useful to report on what proportion of data for each variable was missing. This should also be stated in Table 2 (as additional information below that table).

***Neonatal pre-transport stabilization equipment and medical supplies.***

 Did you collect data on whether the equipment you found was functional and in use for newborn care?

 As the manuscript title suggests, referral infrastructure was a key component of this study. I would therefore suggest that the table in Appendix 1 is moved to the main manuscript.

 Also, what the data in Appendix 1 doesn’t convey is whether the numbers of each item (equipment) listed met the international thresholds for adequacy when linked to the number of sick newborns admitted/referred.

***Referral documentation and communication infrastructure***

 This is very well described and brings out the key deficiencies.

***Neonatal transport resources, drugs, equipment and medical supplies in the ambulance(s)***

 Again, this is very well-written and brings out some very key gaps on the referral process.

**Discussion**

 This has good balanced arguments backed by context-relevant and up-to-date data, but there is no statement about the study limitations.

**Conclusions**

 These are written as definitive and do not account for the limitations of the study. I would recommend revising these to highlight the limitations and need for more further more robust evaluation that these findings will inform.

Is the work clearly and accurately presented and does it cite the current literature?

Yes

If applicable, is the statistical analysis and its interpretation appropriate?

Partly

Are all the source data underlying the results available to ensure full reproducibility?

Partly

Is the study design appropriate and is the work technically sound?

Yes

Are the conclusions drawn adequately supported by the results?

Partly

Are sufficient details of methods and analysis provided to allow replication by others?

Partly

Reviewer Expertise:

Infant and early child health, growth and nutrition. Currently focussing on the post-discharge care of preterm/low birthweight infants in Africa.

I confirm that I have read this submission and believe that I have an appropriate level of expertise to confirm that it is of an acceptable scientific standard, however I have significant reservations, as outlined above.

# Reviewer report: Reviewer response for version 1

**DOI:** 10.21956/wellcomeopenres.20925.r56055

**Published:** 2023-04-21

**Authors:** Susan Niermeyer

**Affiliations:** 1 Colorado School of Public Health, University of Colorado School of Medicine, Aurora, CO, USA

The manuscript examines the characteristics of patients and the attributes of the neonatal referral and transport process for three key hospitals in Nairobi County.  This snapshot of the status of neonatal referral and transport exposes many gaps and highlights the significant morbidity and mortality associated with referral and transport in this context and similar settings in sub-Saharan Africa.  Although descriptive in nature, the analysis uses readily transferable measures which can be assessed in other settings and used to identify points for action.

**Abstract** **:**  The abstract accurately summarizes the results of the study.  In the abstract, it is not entirely clear how referrals to and from the three first-level referral hospitals are accomplished and whether the same ambulance picks up patients from the community/lower-level hospitals *and* transports patients from the first-level referral facility to the tertiary facility.  An alternative, in which parents bring their newborn infants to the first-level facility in a variety of vehicles, occurs commonly.

**Introduction:**  The introduction might benefit from a schematic that shows the relationship of the 3 study sites to the tertiary center and provides more information on the community and lower-level sources of referral to the first-level referral centers.  This schematic could also identify the in-facility, ambulance, and communications infrastructure and services that were assessed as part of the study.  It is important to understand the mode of transport from the community and lower-level facilities to the first-level referral centers and have a sense of the number of sites contributing referrals to each hospital.

**Methods:**  The respiratory support capacity of the maternity hospital neonatal care unit should be specified, much as it is described for Hospitals 2 and 3.  It is unclear whether the Clinical Information Network is a feature of routine data collection in the public hospitals or exists only to support research.

 In the discussion of sampling and sample size, there is reference to 13 months of data; however, the study duration was 12 months.

**Results:**  The results section discusses the characteristics and outcomes of babies referred into the first-level referral hospitals but provides little information on the outcomes of those referred from first-level hospitals to the tertiary facility.  Were these outcomes known?  If not, this is another important point to make.

 The description of neonatal transport resources in the ambulance(s) is somewhat confusing.  This paragraph makes reference to the internal component including transfers into the newborn unit from the labor ward.  Later, there is mention that intrahospital transfers not part of a referral in or out were not assessed.

 The second paragraph of this section states “None of the equipment, drugs, and supplies for newborn care…were available in the ambulances.”  However, Table 3B indicates that oxygen, nasal cannula and face mask were available in the ambulance of Hospital 3.

**Discussion:**  The discussion states that “disease episodes [of referrals] do not differ from those inborn”; however these comparisons were not presented in the results section.  In the discussion of the very high rate of hypothermia among infants referred into the three hospitals, it is still unclear what means of transport were involved at this stage.

 The discussion highlights actionable points well.  It does not address potential limitations of the retrospective, observational design.

**Conclusions:** The conclusion is supported by the findings of the study.  Suggestions regarding the next steps might be labeled as “future directions”.

**References:**  References are current, pertinent, and completely cited.  The references focus on sub-Saharan African comparisons, not a wider comparison with Latin America and/or Asian experience.

**Figures and Tables: **Appendix 1 has several entries that require further definition, including: 1% TEO, oral ampicillin vs. oral amoxicillin, IVF Giving Set, Normal Saline vs. IV Normal Saline, IV solutions vs. Glucose 10%.  There is no mention in the inventory of other equipment and supplies necessary to administer oxygen safely to newborns, such as neonatal flowmeters, blenders, nasal cannulae, face mask, and head hood.  It is unclear whether consumables necessary for the safe operation of incubators, warmers, and pulse oximeters were assessed as part of the primary device.

Is the work clearly and accurately presented and does it cite the current literature?

Partly

If applicable, is the statistical analysis and its interpretation appropriate?

Yes

Are all the source data underlying the results available to ensure full reproducibility?

Yes

Is the study design appropriate and is the work technically sound?

Partly

Are the conclusions drawn adequately supported by the results?

Yes

Are sufficient details of methods and analysis provided to allow replication by others?

Partly

Reviewer Expertise:

neonatal resuscitation, global newborn health

I confirm that I have read this submission and believe that I have an appropriate level of expertise to confirm that it is of an acceptable scientific standard, however I have significant reservations, as outlined above.
